# Annotations

**Published:** 1903-05-30

**Authors:** 


### Annotations.

### Laughter as a Therapeutic Agent.

Laughter has for long been credited with health-giving properties. "Laugh and grow fat" has proved a favourite prescription from time immemorial. And certainly the mental condition which is a necessary precursor of true laughter must be considered as not only rich in alleviating and reparative influence, but also an important factor in the establishment and maintenance of prophylactic powers. Professor Sully in his recently published and most charming monograph "On Laughter" describes, in a manner peculiarly fascinating, its forms, causes and development, and indicates its value in the service of man. We would suggest a study of some such volume as this to many of the lugubrious physicians who are mystified by the all too evident fact that, professionally speaking, they are accredited as "failures." No serious student of the human, and certainly no one desirous of benefiting his fellow creatures, can afford to neglect the immense service which laughter is capable of rendering in rectifying, renovating, and restoring distressed minds and deranged bodies. In the evolution of man laughter plays a part of no little importance, and its withdrawal robs human development of one of its most valuable up-building agencies. It is well that every doctor and every nurse, whether he or she knows aught of the science of laughter or not, should at at all events not be wanting in the proper practice of the soul-quickening and body-soothing art.

### A Hospital Myth.

Free, voluntary work in the cause of suffering was formerly confined mainly to members of the medical profession. A few years ago it commenced to be fashionable, and to-day personal service for the sick has become a definite motive in our daily life. It has found expression in the announcement of their Majesties' intention to attend the services at St. Paul's on Sunday, June 7th, when the Hospital Sunday collection will be made in the metropolitan Cathedral. The crusade of personal service is spreading in a marked degree through every section of the community, and we are glad to take this opportunity of expressing once more our sense of indebtedness and appreciation to our colleagues in the daily press, who generously find room for an amount of "copy" on behalf of the hospitals every year, which could never be published did they not recognise the privilege of taking part in the far-reaching work of ministering to the sick. And here we would say, that we hope neither speakers nor writers will henceforth publish statements broadcast, to the effect, that the voluntary hospital system is in peril for want of funds. That statement is not only inaccurate, but distinctly contrary to the fact. During the year 1902, for instance, when the income and expenditure under all heads of all the hospitals and medical charities of London which participate in the Hospital Sunday Fund are taken in the aggregate, we find, that the total expenditure was £1,161,000 and the total revenue £1,158,000, proving last year's actual deficiency to have been but £3,000 sterling. The huge deficiencies claimed are manufactured by ignoring legacies, which are a regular, constant, and on the whole increasing source of revenue year by year. In fact the voluntary hospitals of London are practically financed, thanks greatly to the increased interest now taken by individual citizens in the work ot the hospitals, largely owing to the efforts of King Edward's Hospital Fund and the League of Mercy, both of which organisations we owe mainly to the interest taken in these institutions by the King himself.

### Women and Whisky.

The annual report of the Dalrymple Home for Inebriate Women at Rickmansworth raises some interesting questions as to the temptations that lead women to inebriety. Of the 679 cases which have been treated at that home since it was established 19 years ago it is reported that 241 are doing well, and 100 have improved. Of the remainder, 203 show no improvement, 9 have become insane, 48 are dead, and 78 have been lost sight of. Thus rather more than half the patients have benefited more or less by their stay in the retreat. But a very important point is raised by the statistics as to what form of alcohol led to the formation of the habit of indulgence which brought these women to Dalrymple House. Of the 679, only 11 were beer drinkers, and 36 habitually drank wine. The rest became drunkards through taking spirits, and especially whisky. Now, while of late years doctors have very largely avoided recommending alcohol to their patients at all, when they do prescribe any it is whisky. If a man or woman of middle age feels twinges of gout or rheumatism, the medical attendant very often advises the giving up of the glass of sherry or claret which the patient has been in the habit of taking, and substituting a little whisky and soda water. From the profession the public have learned to believe that whisky is the "safest" of all spirits, with results very advantageous to distillers. These figures which tell the temptation to which 600 inebriate women succumbed make one doubt if the advice is equally good for the public. It is true that the doctors recommend only "a little" stimulant, and may even specify the quantity, but they have no guarantee that their limitations are adhered to. The warmth of the spirit not merely induces a pleasant sense of well-being, but may even give a temporary relief from pain. This tempts the patient to return to it when the pain comes back. The doctor is not told of this extra indulgence, and the patient consoles herself with the notion that whisky is "so safe." At the menstrual periods even young women are tempted to take spirit to relieve pain, and women who have been temperate all their lives often become inebriate at the climacteric. Neither wine nor beer gives the temporary alleviation sought, but spirits do, and the notion that whisky is not conducive to the development of any disease is a temptation to take it. This is a point which medical men might do well to consider. They may save their patients from falling if they can assure them that whisky drinking will cause some disease. But it is of no use to warn them of the risk of acquiring the habit of inebriety. That only gives offence, and does little good, for no one realises their susceptibility to that temptation until, unhappily, it is too late.

